# Ligandability at the Membrane Interface of GPx4 Revealed through a Reverse Micelle Fragment Screening Platform

**DOI:** 10.1021/jacsau.4c00427

**Published:** 2024-06-26

**Authors:** Courtney L. Labrecque, Brian Fuglestad

**Affiliations:** † Department of Chemistry, Virginia Commonwealth University, Richmond, Virginia 23284, United States; ‡ Institute for Structural Biology, Drug Discovery and Development, Virginia Commonwealth University, Richmond, Virginia 23219, United States

**Keywords:** protein NMR, glutathione peroxidase 4, fragment-based drug discovery, peripheral membrane protein, protein–ligand interactions, protein–membrane interface

## Abstract

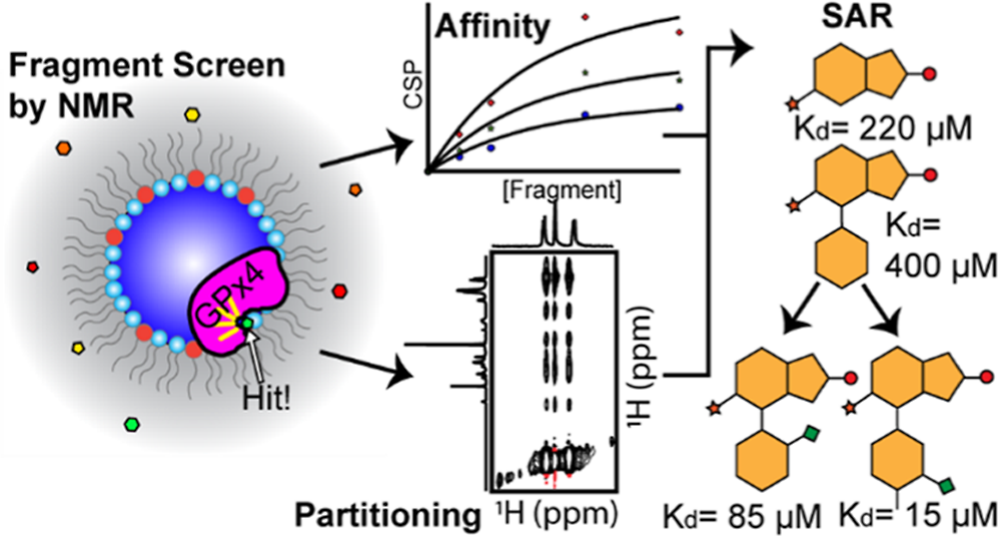

While they account for a large portion of drug targets, membrane proteins present a unique challenge for drug discovery. Peripheral membrane proteins (PMPs), a class of water-soluble proteins that bind to membranes, are also difficult targets, particularly those that function only when bound to membranes. The protein–membrane interface in PMPs is often where functional interactions and catalysis occur, making it a logical target for inhibition. However, protein–membrane interfaces are underexplored spaces in inhibitor design, and there is a need for enhanced methods for small-molecule ligand discovery. In an effort to better initiate drug discovery efforts for PMPs, this study presents a screening methodology using membrane-mimicking reverse micelles (mmRM) and NMR-based fragment screening to assess ligandability at the protein–membrane interface. The proof-of-principle target, glutathione peroxidase 4 (GPx4), is a lipid hydroperoxidase that is essential for the oxidative protection of membranes and thereby the prevention of ferroptosis. GPx4 inhibition is promising for therapy-resistant cancer therapy, but current inhibitors are generally covalent ligands with limited clinical utility. Presented here is the discovery of noncovalent small-molecule ligands for membrane-bound GPx4 revealed through the mmRM fragment screening methodology. The fragments were tested against GPx4 under bulk aqueous conditions and displayed little to no binding to the protein without embedment into the membrane. The 9 hits had varying affinities and partitioning coefficients and revealed properties of fragments that bind within the protein–membrane interface. Additionally, a secondary screen confirmed the potential to progress the fragments by enhancing the affinity from >200 to ∼15 μM with the addition of certain hydrophobic groups. This study presents an advancement of screening capabilities for membrane-associated proteins, reveals ligandability within the GPx4 protein–membrane interface, and may serve as a starting point for developing noncovalent inhibitors of GPx4.

## Introduction

Membrane proteins (MPs) account for around 23% of the proteome and over 60% of the current drug targets.^1^ Peripheral membrane proteins (PMPs) are a diverse subcategory of water-soluble, membrane-associated proteins that interact, often reversibly, with membranes via electrostatic, hydrophobic, or specific lipid headgroup interactions.^2,3^ PMPs have emerged as a prominent category of drug targets for a variety of disease processes.^4^ Despite the great need for inhibitor development for integral membrane proteins and PMPs, many roadblocks remain. While integral membrane proteins do present a challenge for certain inhibitor design approaches, high-throughput screening (HTS) and some structure-based design methods have had success, with the majority of small-molecule inhibitors confined to aqueous-exposed binding sites.^1,5^ Recently, attention has shifted toward inhibitors that bind within the protein–membrane interface, with this mode of inhibition becoming more prominent.^6^ Despite the recognition of the importance of drug–lipid interactions, protein–lipid interfaces are often overlooked during the initial stages of screening and rational drug design due to challenges in housing proteins in a membrane-like environment that is also compatible with screening.^7−9^

Like integral membrane proteins, PMPs are also challenging drug targets. This class of protein is oftentimes only active when bound to the membrane, with the water-solubilized state being inactive.^4^ For many PMPs, the membrane interface generally represents the functional site of the protein that should be targeted for inhibition.^4^ Despite this, PMPs are typically screened in bulk aqueous conditions due to the readily available methods and the retained stability of this class of proteins in the absence of a membrane.^4,10^ To date, many PMPs are considered “undruggable”, meaning the target is considered too difficult to probe by standard methods of screening.^1,11,12^ A hallmark of druggability is the presence of a solvent-accessible hydrophobic pocket, often but not exclusively, represented by an active site for enzyme targets.^1,13^ On the other hand, when the target is a membrane-associated protein, active sites do not necessarily translate to solvent accessible.^1^ To access a specific binding site of a membrane protein that is exposed to the lipid phase, the small molecule will first have to partition into or onto the bilayer before engaging binding sites on the target.^6,7^ The ability of proteins to bind small molecules is termed ligandability, which is a prerequisite for a target to be considered druggable.^14,15^ Ligandability of membrane-associated proteins at the membrane interface is an underexplored area, meaning it is mostly unknown the types of ligands that could target protein–membrane interfaces.^6^

A powerful approach for inhibitor discovery and design, fragment-based drug discovery (FBDD), shows great promise for the discovery of new modes of inhibition. FBDD allows discovery of novel compound classes since the fragment libraries are typically optimized for chemical diversity, and nearly infinite possibilities of combinations provide ample pathways to previously unknown inhibitor types.^16,17^ FBDD is based on initial discovery of very small molecules (<300 Da) that bind to a protein target.^18^ Fragment screening is a useful approach for assessing whether a protein target is ligandable and reveals the initial building blocks for FBDD approaches.^14,19^ Due to the small size, two or more fragment hits may be linked, fragments may be grown, or elements of fragments can be combined to yield a larger, higher affinity compound.^20^ Due to the inherently weak nature of fragment binding, biophysical screening methods are almost always required.^20−22^ Additionally, optimal elaboration generally requires structural information, which can be provided by protein-detected NMR as well as crystallography.^23,24^ Widely used membrane models for biophysical methods include micelles, bicelles, liposomes, and nanodiscs, with the recently developed membrane-mimicking reverse micelles (mmRMs) as a promising addition for protein NMR experimentation.^25,26^ While liposomes are too large, micelles, bicelles, and nanodiscs are all amenable to protein NMR and have provided invaluable insights into protein–membrane interfacial interactions.^27^ However, these models have some drawbacks that may limit their practicality in protein-detected NMR fragment screening approaches. Bicelles and nanodiscs are very large assemblies, making them less practical for screening due to the need for deuteration of the detergent and protein for even modestly sized proteins.^28^ Additionally, nanodiscs require preparation of a scaffold protein, an increased burden for screening which utilizes large numbers of samples.^29^ Micelles, while smaller, are generally constructed of artificial detergents that can distort the protein structure and limit sample stability.^5,30,31^ Alternatively, reverse micelles (RMs) have not only proven to be useful for a variety of biophysical protein NMR studies but they have been shown to be compatible with fragment screening of water-solubilized proteins.^32−35^ The recent development of mmRMs promises to extend the biophysical and fragment screening approaches developed in RMs to membrane-associated proteins.

The protein–lipid bilayer interface is a largely untapped avenue for drug discovery. To further complicate discovery efforts, libraries that are utilized for HTS screening campaigns tend to be populated with known inhibitors, natural products, and their analogues, which would limit the discovery of unknown classes of molecules.^36−38^ As membrane proteins become more prominent as drug and inhibitor targets, the need for novel classes of compounds is apparent. A fragment screening approach allows sampling of a large chemical space while avoiding the bias present in high-throughput libraries.^23,39^ Additionally, exploration of protein–membrane interface ligandability is worthwhile due to the recent attention to this mode of inhibitor binding.^6^ We used glutathione peroxidase 4 (GPx4) as our proof-of-principle system, a lipid hydroperoxidase that functions at cellular membranes to reduce lipid hydroperoxides.^40−42^ GPx4 is a highly sought-after drug target due to its regulation of ferroptosis, an iron-dependent and nonapoptotic form of cell death^43−45^ which has shown promise in eliminating therapy-resistant cancer cells known as persister cells.^46−48^ GPx4 is a difficult target due to its lack of a deep active-site pocket, and it functions as a lipid hydroperoxidase only in its membrane-bound state.^49^ The warheads currently used to target GPx4 are chloroacetamides, namely, (1*S*,3*R*)-RSL3 (RSL3) and ML162, and masked electrophiles such as ML210. While masked electrophiles like nitrile oxides are prodrugs with some improved selectivity compared to the low stability and high promiscuity of the chloroacetamide probes,^49^ moving away from these covalent warheads altogether would avoid the disadvantages with this type of reactive electrophile.^50^ The footprint of GPx4 against its membrane is large and highly cationic^51,52^ precluding a strategy aimed at blocking membrane binding.^53,54^ In contrast, functional residues are localized to small, potentially ligandable regions of the protein within the membrane interface, with the cationic patch implicated in lipid binding and the catalytic site for enzymatic activity.^51,52^ A noncovalent inhibition strategy may target this lipid binding region to prevent engagement with substrates. We sought to screen the functional membrane-bound state of GPx4 for fragment ligands, with the ultimate goal of assessing ligandability and unveiling potential building blocks for inhibitors that block lipid binding or catalysis in the membrane–protein interface.

To undertake a fragment screen of GPx4, we selected the most stable and suitable membrane model, which we found to be mmRMs composed of a mixture of 1,2-dilinoleoyl-*sn*-glycero-3-phosphocholine (DLPC) and *N*-dodecyl phosphocholine (DPC) at concentrations of 37.5 mM each. This formulation has been previously demonstrated to encapsulate multiple PMPs, demonstrating that this method may be applied to a range of membrane interacting proteins.^25,26^ A successful screen of the protein was conducted, identifying nine fragment hits all with an apparent affinity less than 1 mM, demonstrating ligandability of the membrane-bound form of GPx4. The fragment hits spanned a range of partitioning coefficients and behavior. Importantly, they lacked significant binding to the water-solubilized form of GPx4, even at extreme concentrations. Finally, while the fragments weakly bind, secondary screening experiments were performed to demonstrate structure–activity relationships (SARs) and the possibility to expand and enhance the small fragments toward a noncovalent inhibitor of GPx4. This approach promises to be a powerful tool in the fragment screening arsenal, has revealed fundamental properties of ligands that bind within protein–membrane interfaces, and has uncovered noncovalent fragments for GPx4 which may be starting points for a new class of inhibitors.

## Results and Discussion

### Membrane Model Selection

A variety of membrane models are available for the study of MPs and their relevant interactions, leaving us with the challenge of finding the best model for fragment screening. Since protein NMR is one of the most preferred methods for fragment screening, we limited our membrane model selection to those that are compatible with this technique. Stability of the membrane model is important for fragment screening to ensure that breakdown or expansion of the model is not misinterpreted as a false positive hit. To ensure the solubility of fragments in aqueous buffer conditions, DMSO is commonly used, though we anticipated that DMSO may interfere with established membrane models. To assess, we measured disturbance to the detergent assemblies upon the introduction of 5% DMSO using dynamic light scattering (DLS). Introduction of 5% DMSO to DPC micelles increased the size from 4.3 to 5.1 nm (Figure S1a), indicating incorporation of the cosolvent. DHPC/DMPC isotropic bicelles showed that a second population is formed in the presence of 5% DMSO around 5.2 nm accounting for 20% of the total population, which indicates breakdown of the assembly (Figure S1b).^55^ While nanodiscs are excellent membrane models, their use in fragment screening by protein NMR is less practical due to their large size, requiring deuteration of lipid components and the requirement to express and purify a scaffold protein in order to construct. For these reasons, we sought another alternative.

In contrast to micelles and bicelles, RMs are solubilized in a bulk alkane phase, including a hexanol cosurfactant. The surfactants and cosurfactants form a spherical shell surrounding a nanoscale pool of water containing the protein.^34^ Recently developed mmRMs constructed from a mixture of DLPC and DPC allow embedment of PMPs into the phosphocholine-rich inner shell as they would within a membrane.^25^ Hydrophilic small molecules are known to be highly soluble within the water core of RMs, while the alkane–hexanol-mixed solvent enables solubilization of more hydrophobic small molecules.^32^ Together, the mixed solvent system promises to allow direct delivery of fragments to the mmRM system without the need for DMSO and with minimal perturbation of the membrane model. Fragments are generally screened in mixtures to increase throughput; therefore, compatibility with fragment mixtures is essential. To confirm, we solubilized 10 mixtures of 10 fragments in separate mmRM samples, without DMSO. Of the 10 mixtures, only 1 mmRM sample had visually insoluble fragments. The mmRM size remains relatively constant with the addition of 10 fragments as observed by DLS (Figure S1d). A complementary experiment was performed with DPC micelles and the same 10 mixtures without DMSO. In contrast with mmRMs, 4 out of 10 mixtures were observed to have insoluble aggregation in the DPC micelle samples. Size was assessed with DLS, showing similar variance as fragments with mmRMs (Figure S1d); yet, the lower proportion of fragment solubilization and incompatibility with DMSO reduce the utility of micelles for fragment screening. In addition, mmRMs have other advantages over micelles and bicelles, such as enhanced protein stability compared to micelles, favorable tumbling properties compared to bicelles and nanodiscs, and simplicity of construction compared to nanodiscs.^27^ Importantly, DLPC/DPC mmRMs are known to also encapsulate fatty acid binding protein 4 and phosphatidylethanolamine binding protein 1 in their membrane-bound state.^25,26^ These proteins and GPx4 are structurally and functionally distinct, demonstrating that a breadth of PMPs may be encapsulated within mmRMs for study, with only minor protein-specific optimizations needed, such as water content and hexanol concentration.^25,26^ Together, these advantages and enhanced fragment compatibility led us to pursue mmRMs as a platform to fragment-screen membrane-bound PMPs, using GPx4 as our proof-of-principle target.

### Fragment Screening within mmRMs

**Figure 1:**
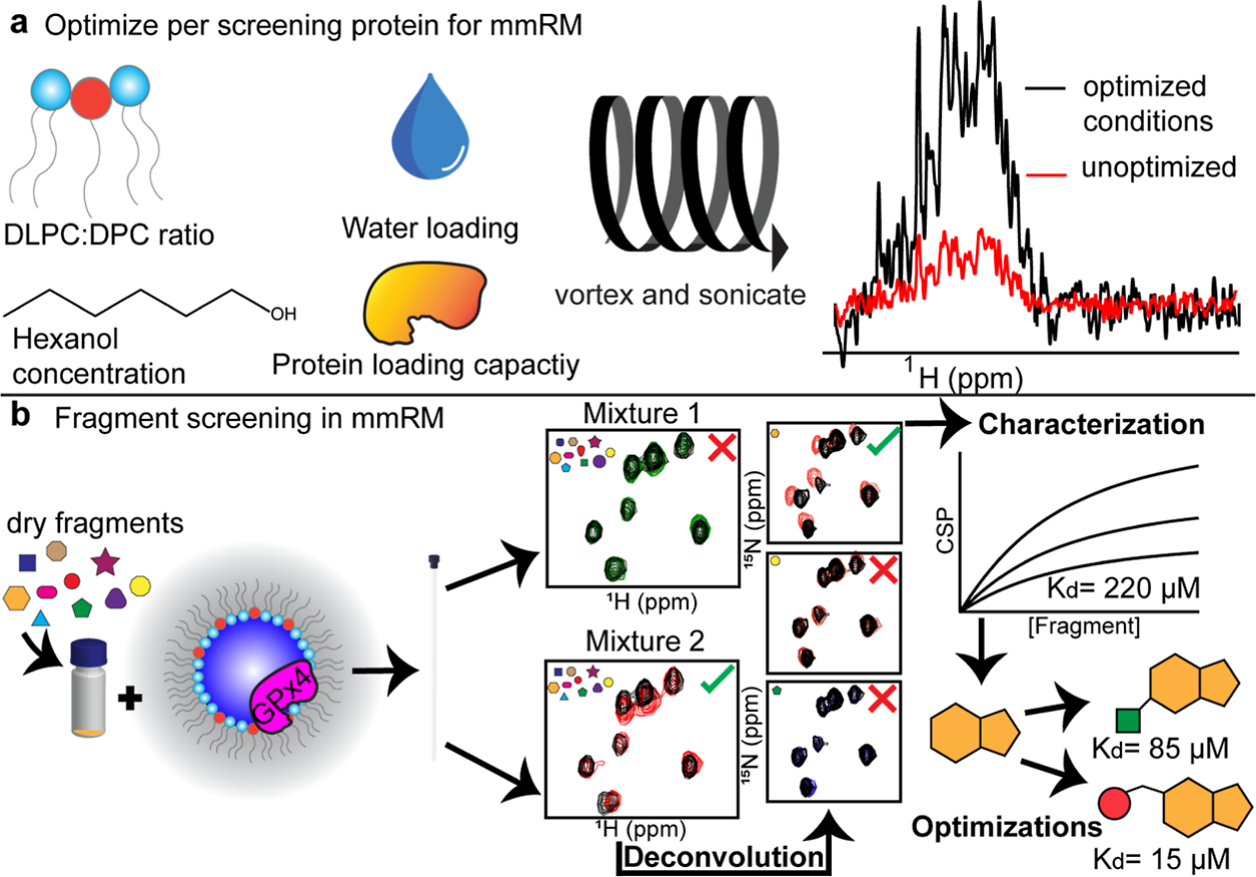
mmRM fragment screening workflow. (a) Parameters such as DLPC/DPC ratio, water loading (water-to-surfactant molar ratio, *W*_0_), hexanol concentration, and protein concentration are optimized prior to the addition of fragment mixtures. (b) Once optimized, fragment mixtures are dried and mixed with preconstructed mmRMs with the protein of interest already encapsulated. Routine fragment mixture screening and deconvolutions by protein-detected NMR are performed to isolate hits for the protein of interest. Characterization of hits was performed by NMR titrations to extract apparent *K*_d_s. Finally, optimizations of the fragments may lead to enhanced binders for the protein of interest.

With mmRMs selected as our membrane model, we undertook a protein-observed NMR-based fragment screen of GPx4. The screen was initially established by optimizing the encapsulation of GPx4 (Figure 1a). Once the mmRM conditions were optimized, a commercial, 1911-member fragment library was screened, which contained fragments that are rule-of-three compliant,^18,20^ were filtered to avoid PAINS,^56^ and were selected to sample broad chemical diversity. Fragments were initially screened at a 4:1 fragment to protein molar ratio in mixtures of 10 to enhance throughput. The fragment to protein ratio selected here corresponds to conditions within standard ranges commonly used for previously reported fragment screens.^20^ After completion of the fragment screen, the hit rate, fragment-induced chemical shifts, and fragment hit affinities suggest that fragment/protein molar ratios from approximately 2:1 to 8:1 are appropriate for this approach. Use of mmRMs eliminated the need for DMSO to solubilize fragments. Encapsulation of GPx4 within mmRMs proceeded as previously reported with adjustments to the buffer.^25^ To deliver the fragments, the entire preconstructed mmRM sample was transferred to the vial containing the predried mixtures. Over 90% of the fragment mixtures were fully soluble in mmRM, with the remaining 10% containing minor insoluble aggregates. Mixtures containing insoluble aggregates were nevertheless screened for fragment binding, with the assumption that fragments that could not be solubilized would not interfere and be misinterpreted as hits. No fragment hits were observed in samples containing insoluble aggregates, confirming that their presence did not inherently lead to false positives. The 191 mixtures of 10 fragments apiece were analyzed by ^15^N-HSQC experiments of GPx4. Any mixtures showing promising chemical shifts in the membrane-interacting surface were flagged as potentially containing a hit (Figure 1b). Fragment members of the 10 hit mixtures showing the most shifting were tested individually at a 4:1 ligand/protein ratio to reveal the identity of the hit within each mixture. To select hits from nonhits, we established chemical shift perturbation (CSP) cutoffs, with 7 out of the 15 observable cationic patch or catalytic site resonances mapped from previous experiments^25^ producing CSPs greater than 0.01 ppm. This hit selection criteria was based on observing CSPs in a significant number of residues in functionally important residues that should be targeted for inhibitor development, and the CSP cutoffs based on typical CSPs expected from fragment binding.^24^ Using these CSP cutoffs, 14 individual fragments were identified as potential GPx4 binders. These 14 fragments were characterized by titration to validate the hit and assess affinity.

### Fragment Validation and Binding Affinity Determination

**Figure 2:**
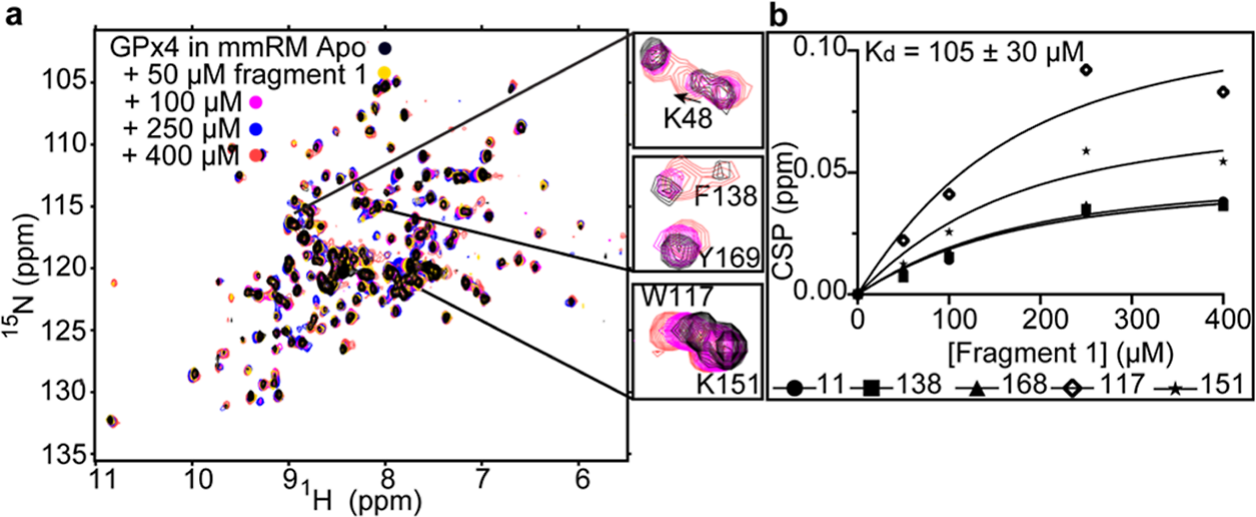
Titration of fragment 1 against GPx4 embedded in a mmRM. (a) Overlay of [^15^N–^1^H] HSQC of GPx4 in the DLPC/DPC mmRM (black) with 50 (yellow), 100 (pink), 250 (blue), and 400 μM (red) of fragment 1. Zoomed panels show example resonances with chemical shift perturbations. For ease of visualization, only apo GPx4 along with 100 and 400 μM fragment 1 is shown in the zoom panels. (b) Plot of representative chemical shift perturbations used for the apparent *K*_d_ extraction for fragment 1 against GPx4. Resonances used for *K*_d_ fitting were selected by identifying the top-sifting resonances at 400 μM fragment 1. These were resonances with a CSP at least 1σ above the average CSP and corresponded to residue numbers 11, 117, 138, 151, and 168. These top shifters were then fit to individual *K*_d_ curves and resonances with fits with a *R*^2^ < 0.85 was removed. The remaining resonances were then included in a global fit to determine the overall apparent *K*_d_.

To assess whether the fragment hits are bound specifically to GPx4 and to calculate affinity, we performed NMR-based titrations. Chemical shift changes that approach saturation in a structurally localized manner target indicate specific binding.^24^ This allows true positive hits to be separated from false positives and weak or nonspecific binders. Additionally, NMR-based titrations allow for the extraction of a binding affinity (*K*_d_). To calculate apparent *K*_d_ values, CSP cutoffs were used to isolate the resonances with most shifting using 1 standard deviation above the average CSP. All 14 fragments identified from the deconvolutions were titrated up to 400 μM or 1 mM if needed. Of the 14, 9 had apparent *K*_d_s less than 1 mM and were identified as hits. Observed here is a 0.47% hit rate with best affinities in the μM range for the membrane-bound form of GPx4 and is consistent with ligandable and potentially druggable targets.^14,57^ The highest affinity fragment, fragment 1, had an apparent *K*_d_ of 105 μM ± 30 (Figure 2a,b). The remaining eight fragment binding curves are shown in Figure S2. The remaining five fragments, 10–14, were ruled out as weaker (>∼1 mM) or nonspecific binders. Weak or nonspecific binding could indicate that fragments partitioning to the mmRM surface and perturb the protein spectrum through a simple change in the chemical environment at the interface, among other possible nonspecific effects. Protein-detected titration curves that approach saturation and are structurally localized indicate that the fragment is indeed binding specifically and directly to the protein, demonstrating the importance of hit validation. We note that the weaker and nonspecific fragments were only eliminated due to interaction modes that are too weak or unproductive for advancement toward inhibitors; yet, binding was nevertheless detected. No true false-positive, noninteracting fragments were observed at this stage which highlights the robustness of the reported approach for detecting and validating fragment hits. Apparent *K*_d_ values are summarized in Table 1. We note here that apparent *K*_d_ values reported in this study account for a total amount of fragment and protein within the mmRM sample and are not corrected for any potential concentrating effect due to localization of fragment to the mmRM surface or water phase.

**Table 1:**
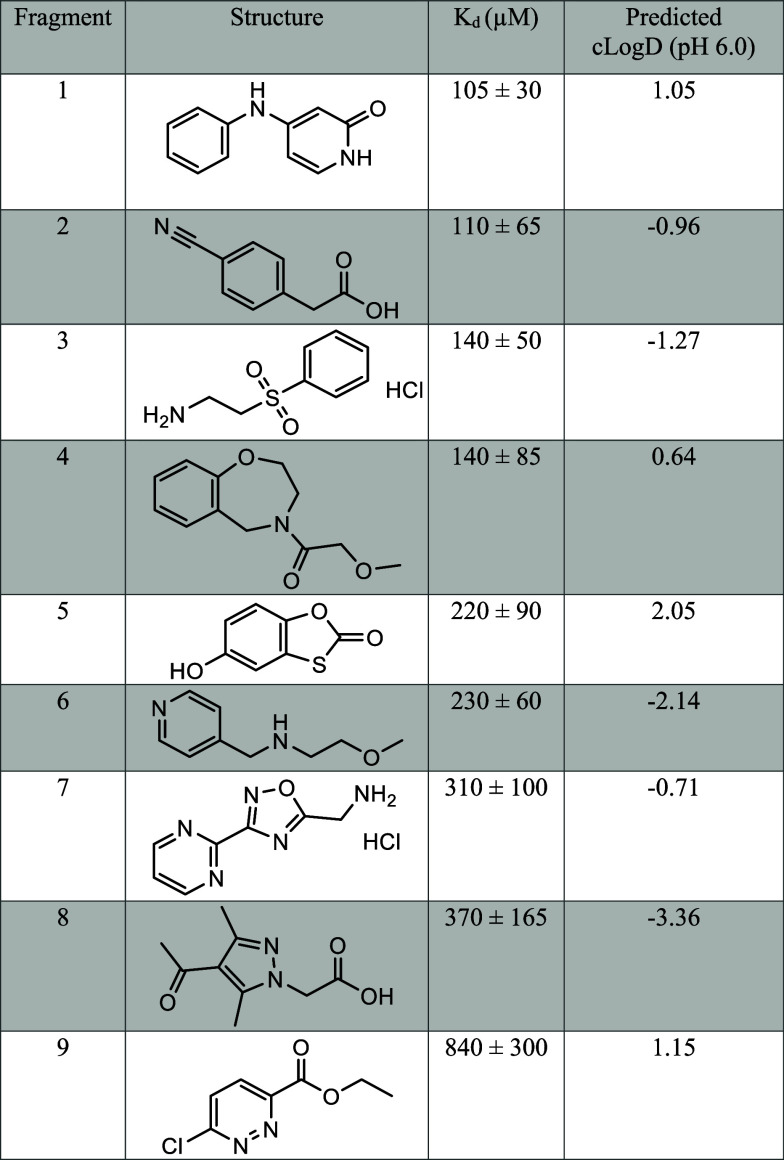
All Fragments Titrated against GPx4 in the mmRM with an Apparent *K*_d_ Less Than 1 mM as Assessed by NMR Titrations^a^

a Standard error of global *K*_d_ fit of all selected residues is reported.

**Figure 3:**
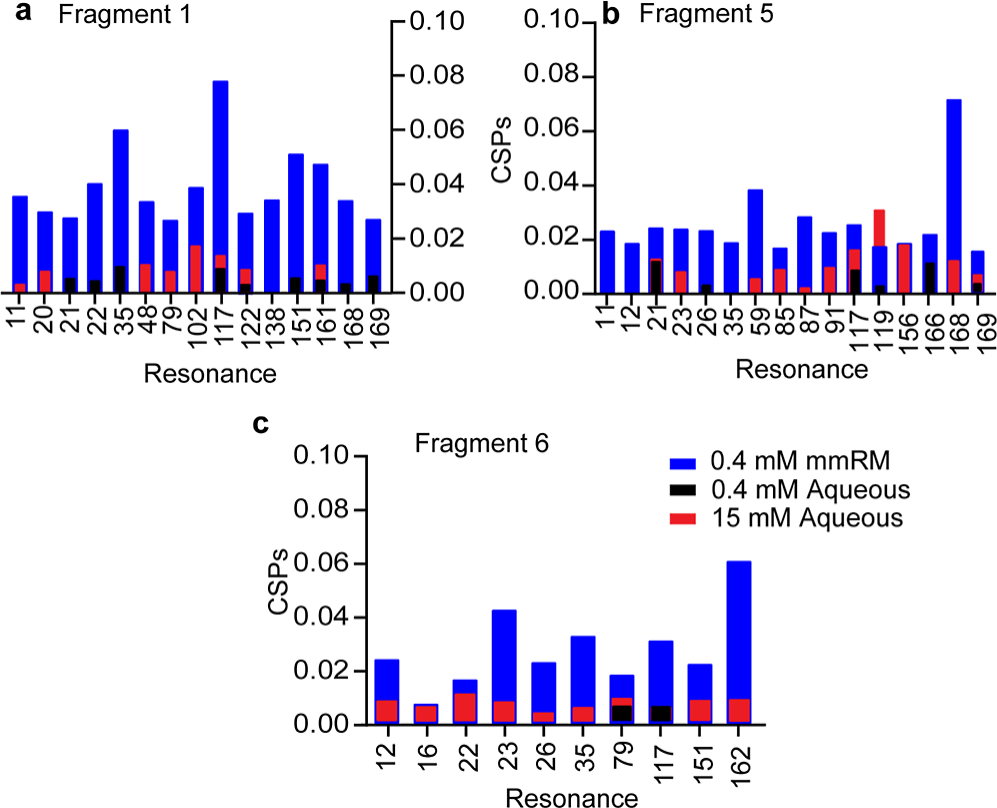
Comparison of hit binding to membrane-embedded versus aqueous GPx4 for (a) fragment 1, (b) fragment 5, and (c) fragment 6. [^15^N–^1^H] HSQC experiments were conducted with 400 μM of fragments 1, 5, or 6. The resonances that produced the greatest CSPs (at least 1σ above the average) for the mmRM experiment (blue) are compared to the corresponding aqueous data. The identical experiment was performed in bulk aqueous conditions (black). 15 mM experiments were also conducted in aqueous conditions (red) to test for very weak binding.

Next, we sought to understand whether fragment hits were solely bound to the membrane-embedded form of GPx4 or could also bind to the aqueous form. We performed aqueous binding experiments with 3 fragment hits with varying hydrophobicities. Of the fragment hits with *K*_d_ < 1 mM, the most hydrophobic fragment (fragment 5, cLog *D* 2.05, 5-hydroxy-1,3-benzoxathiol-2-one) and a hydrophilic fragment with the strongest affinity (fragment 6, cLog *D* −2.14, (2-methoxyethyl)(4-pyridinylmethyl)amine hydrochloride) were selected, along with a compound with an amphipathic structure and only modest hydrophobicity (fragment 1, cLog *D* 1.05, 4-(phenylamino)-1,2-dihydropyridin-2-one). Aqueous GPx4 was combined with 400 μM of either fragments 1, 5, or 6 to directly compare to the mmRM experiments. Comparing the CSPs in aqueous versus mmRM, we observed that the fragment hits do not bind strongly to the aqueous form of the protein (Figure 3a–c). This also suggests that a screen under aqueous conditions would not uncover these fragment hits. Since our fragments have variable degrees of solubility and partitioning, we realized that a hydrophilic fragment like 6 may partition into the water core of the mmRM, essentially causing a concentration effect as observed previously.^32,33^ To rule this out, the concentration of the fragments was increased to 15 mM in an aqueous sample, which would be the effective concentration if all of the fragments partitioned completely into the RM water core. For fragments 1 and 6, neither produced significant CSPs (Figure 3a,c). Fragment 5 does produce some CSPs that could be detected by a soluble screen (Figure 3b). A very high concentration in aqueous conditions (15 mM) was needed to reach CSPs comparable to the mmRM conditions in some resonances, which is much higher than standard screening concentrations of typically ∼100–400 μM. The comparison of fragment 6 at 15 mM in aqueous conditions also demonstrates that observed CSPs are not purely the result of extremely weak binding due to a concentrating effect on the water component of the mmRM. DLS measurements were also collected to check for fragment aggregation in the aqueous conditions.^58^ In aqueous conditions at 400 μM, none of the fragments had any observable aggregation, but at 15 mM fragments 1 and 5, there was a small population of large aggregating species, which was expected considering the cLog *D* values. Inspection of ^1^H NMR spectra confirmed that the aggregation observed at high concentration of fragments 1 and 5 is a small portion of the overall fragment concentration and that most of the fragment is in solution. A lack of observed binding of these fragments to protein highlights that they do not bind to the water-solubilized state of GPx4, with binding of the hydrophobic and amphipathic fragments driven to the membrane interface. This is possibly due to a conformational change upon membrane binding, which is suggested by the very large chemical shift changes in the interfacial region when GPx4 binds to membrane models.^25,26,52^

**Figure 4:**
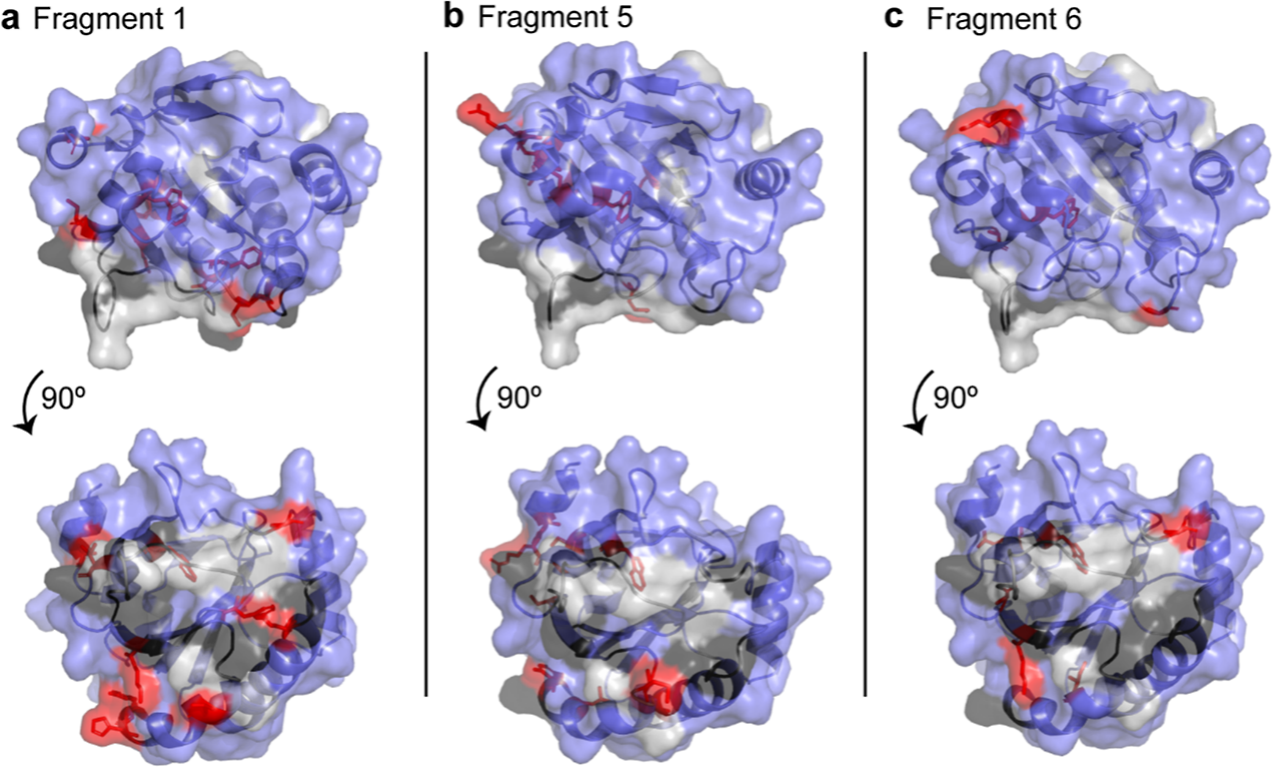
Mapped chemical shift perturbations for the membrane interacting residues of GPx4 with (a) fragment 1, (b) fragment 5, and (c) fragment 6 are displayed with red sticks. Residues that are unassigned in the mmRM are displayed in light gray, residues with little or no shifting are shown in blue, and other membrane interacting residues are in black.

To visualize the fragment binding sites, the top shifting resonances in the membrane interface by CSP analysis for fragments 1, 5, and 6 were mapped onto the crystal structure of GPx4. Both fragments 1 and 5 have several shifting resonances in the membrane interface, indicating that the fragments are able to partition into the mmRM membrane model to bind the protein (Figures 4a,b and S3a,b). We note that due to some line-broadening in the membrane interface of GPx4, not all interface residues are observable. For fragment 1, the significantly shifting residues, A11, K48, G79, W117, I122, F138, K151, V161, H168, and Y169, were within or neighboring the cationic and catalytic sites of GPx4 and the membrane interface.^25^ For fragment 5, shifting residues were similarly A11, R12, W117, W119, C148, M156, L166, and Y169. Fragment 6 seems to have less interaction with the protein at the membrane interface, which could potentially be a result of the overall hydrophilicity of the fragment in comparison to fragments 1 and 5 and binding to the water-exposed allosteric site (Figures 4c and S3c).^59^ Some membrane interface residues did display shifting, with G79, W117, G126, K151, and I162 having the most. Regardless, the presence of the membrane model was necessary for the fragment interaction as observed in Figure 3c. A better understanding of fragment partitioning capabilities could lead to enhanced GPx4 targeting and therefore fragment optimization and elaboration.

### Partitioning Properties of Fragments in mmRMs

**Figure 5:**
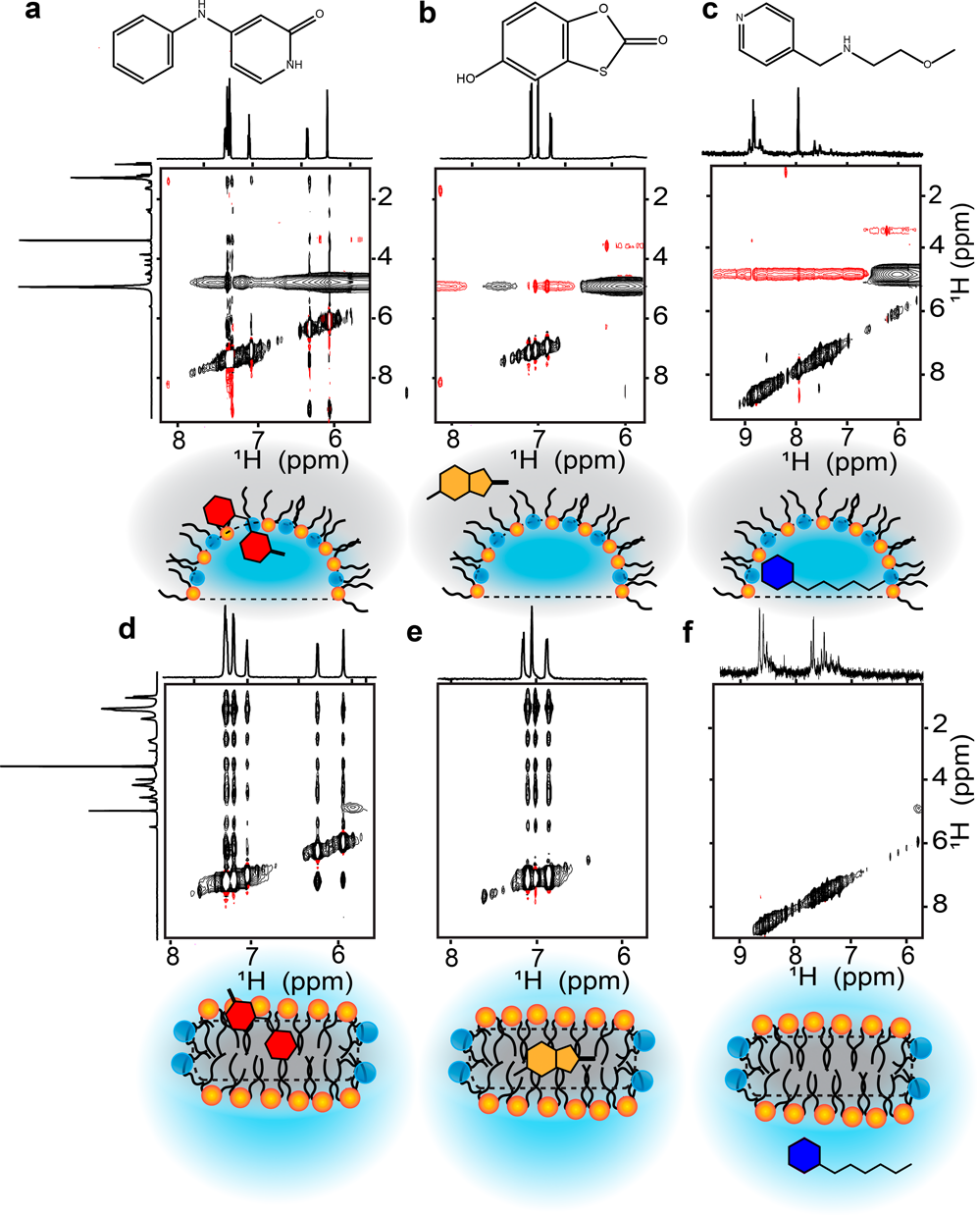
Zooms of [^1^H–^1^H] NOESYs of the three fragments show variable partitioning in the mmRM. (a) Most amphipathic fragment, fragment 1, shows clear interaction with the surfactant shell of the mmRM as well as with water. (b) Fragment 5, the most hydrophobic fragment, does not show any clear NOEs, indicating that the fragment may be entirely residing in the pentane phase in the absence of GPx4. (c) The most hydrophilic fragment, fragment 6, does not have any NOEs to the surfactant shell and may be fully residing in the water core of the mmRM. Positive contours are shown in black, and negative contours are shown in red. Zooms of [^1^H–^1^H] NOESYs of the three fragments in a DMPC/DHPC bicelle are shown to further demonstrate partitioning. All bicelle samples were in D_2_O to reduce the water signal to better observe NOEs. (d) Fragment 1 has interactions with the surfactant shell of the bicelle similarly to the mmRM, but (e) fragment 5 is within the bicelle core. (f) Fragment 6, now in a completely D_2_O phase, still does not have any observable NOEs. Cartoon representations have been included for partitioning visualization.

Interestingly, the 9 validated hits had varying predicted partitioning values (cLog *D*, pH = 6.0) between −3.36 and 2.05, spanning from hydrophobic to hydrophilic (Table 1). We sought to understand the potential membrane partitioning of these fragments by evaluating their behavior in mmRMs, again using fragments 1, 5, and 6 as our benchmarks. To evaluate fragment partitioning in the mmRM system, 2D ^1^H–^1^H NOESY experiments were performed, which report on the spatial proximity of intra- and intermolecular nuclei. Initial experiments demonstrated that 5 mM fragment is needed to clearly observe intermolecular NOEs. The mmRMs were constructed from deuterated pentane and hexanol to reduce solvent signal and associated NOESY artifacts, which were still present, but NOESY measurements were possible.^60^ Our moderately hydrophobic fragment, fragment 1 with a cLog *D* of ∼1, shows clear NOE cross-peaks to the surfactant shell, in particular to the surfactant tails and headgroup, and potentially with water (Figures 5a and S4a). Fragment 5 was the most hydrophobic fragment hit from this screen with a predicted cLog *D* of ∼2. The 2D NOESY reveals that the majority of the fragment must reside in the alkane phase of the reverse micelle since there does not appear to be any relevant cross-peaks between the fragment and the water or the surfactant shell (Figures 5b and S4b). A lack of NOEs to pentane is due to the necessity of using deuterated pentane for artifact reduction and attempts to directly observe solvent NOEs failed. The most hydrophilic fragment, fragment 6 with a predicted cLog *D* of about −2, is expected to predominantly have intermolecular interactions with the water phase of the mmRM (Figures 5c and S4c). However, interference from the water peak introduced streaking and precluded the visualization of clear cross-peaks.

To confirm partitioning behavior, we tested fragments in DHPC/DMPC bicelles, which use a bulk aqueous solvent, and the hydrophobic phase is confined to the bicelle interior. To successfully collect NOESY data, the sample solvent was composed of 100% D_2_O. As expected, fragment 1 partitions to the bicelle, similarly to the mmRM (Figures 5d and S5a). NOEs between fragment 5 and all components of the surfactants indicate full partitioning into the hydrophobic core of the bicelles (Figures 5e and S5b). For fragment 6, no NOE cross-peaks to bicelle surfactants were observed (Figures 5f and S5c), and no water cross-peaks were observed due to the necessity of 100% D_2_O to reduce artifacts. This result confirms that fragment 6 prefers to reside in the water core of the mmRM and the aqueous solvent in the bicelle system. Together, the results highlight the partitioning behavior of fragments that bind to GPx4. Hydrophilic, hydrophobic, or fragments that partition to the surfactant shell are all capable of binding to the membrane-bound state of GPx4 and may be detected using this method.

### Enhanced Affinity through Fragment Optimization

**Figure 6:**
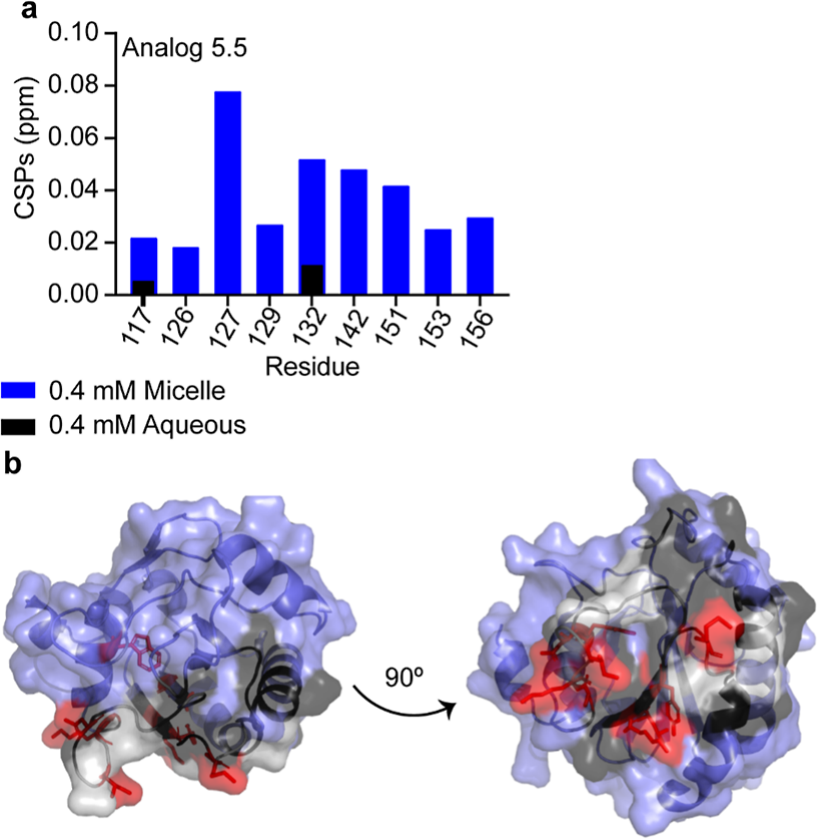
Analogue 5.5 in DPC micelles. (a) Top-shifting resonances from [^1^H–^15^N] HSQC experiments were identified by calculating the CSP between GPx4 bound to DPC micelles with 0.4% DMSO and GPx4 bound to DPC micelles with 400 μM analogue 5.5 an 0.4% DMSO. CSPs at least 1σ above of the average were isolated as the top shifters (blue bars). CSPs from the comparable aqueous experiment that consisted of apo GPx4 with 1.5% DMSO and GPx4 with 400 μM analogue 5.5 and 1.5% DMSO are shown in black bars. The increased DMSO was required for full solubilization in these conditions. (b) Top-shifting resonances were then mapped on a crystal structure of GPx4 with the membrane interface in DPC micelles shown in black, residues with little or no shifting are shown in blue, missing resonances in gray, and analogue interacting residues shown with red sticks.

To investigate whether or not these fragments could be built upon and progress to higher affinity binders and potentially inhibitors, a secondary screening approach was used to find analogues of the original fragments. A fragment growing approach was followed due to the high success rate of improving fragment hits.^61,62^ Fragment growing improves binding affinity, or other properties, with the addition of different substitutions and/or expansions.^20^ Commercially available analogues of fragments 1 and 5 were found by searching the ChemBridge (San Diego, CA) catalog for analogues with a 60% similarity for fragment 1 and a 70% similarity for fragment 5. A subset of analogues for each fragment was purchased and tested. These two fragments were the main focus for fragment progression due to their observed ability to partition into bilayers and bind to inhibitory regions of membrane-bound GPx4. Fragment 6 was not investigated further since it resides mainly in the water core of the mmRM model, and while interesting, it falls outside of our focus for this study. While all the five selected fragment 1 analogues produced reasonable CSPs in the membrane interface of GPx4; none produced titration curves that indicated enhanced binding (Table S1). This result led us to believe that commercially available analogues alone would not lead to the productive development of this specific fragment. Alternatively, for fragment 5, a clear progression of binding affinity and structure optimization became apparent from titrations with compounds containing a phenyl and additional substitutions around the ring, namely, mono- or dichloro or methyl substitutions in the para- or meta-positions (Table 2). Interestingly, addition of the phenyl ring alone reduced affinity, and the presence of at least one chloride was necessary to enhance the affinity. Of the seven analogues of fragment 5 selected for titrations, five fit to apparent *K*_d_s lower than the original fragment, ranging from 15 to 85 μM. Additionally, all of the analogues with an enhanced affinity were more hydrophobic than the original fragment. Meaning, as a whole, these analogues would also partition into membranes efficiently. Addition of hydrophobic groups is a suggested strategy to enhance the affinity of inhibitors to membrane proteins,^6^ which is reflected here. To validate this conclusion, analogue 5.5, which has the highest *c* Log *P* of 4.90 with a high affinity of ∼30 μM, was selected for experimentation in DPC micelles and again in bulk aqueous conditions. The parallel experiment was also completed in mmRMs (Figure S6a,b), with 250 μM analogue 5.5. These experiments will reveal whether analogue 5.5 can target the protein in a membrane model but will not be an effective binder in the absence of the membrane. As expected, reasonable CSPs were observed in the DPC micelle experiment in the membrane interacting interface of the protein, while the comparative aqueous experiment showed little to no shifting that would indicate that the analogue was binding to the protein (Figure 6a,b). DPC micelles have somewhat less line-broadening in the membrane interface compared to the mmRM, allowing a more complete structural map of analogue binding. While not ideal for screening conditions, other membrane models, such as DPC micelles, can be useful for characterizations of some individual hits. Altogether, these results show that the original fragments have the potential to be developed into higher-affinity binders of GPx4. This approach could lead to a noncovalent inhibitor for GPx4 or other similar PMP targets that have thus far proven elusive for noncovalent small-molecule inhibition.

**Table 2:**
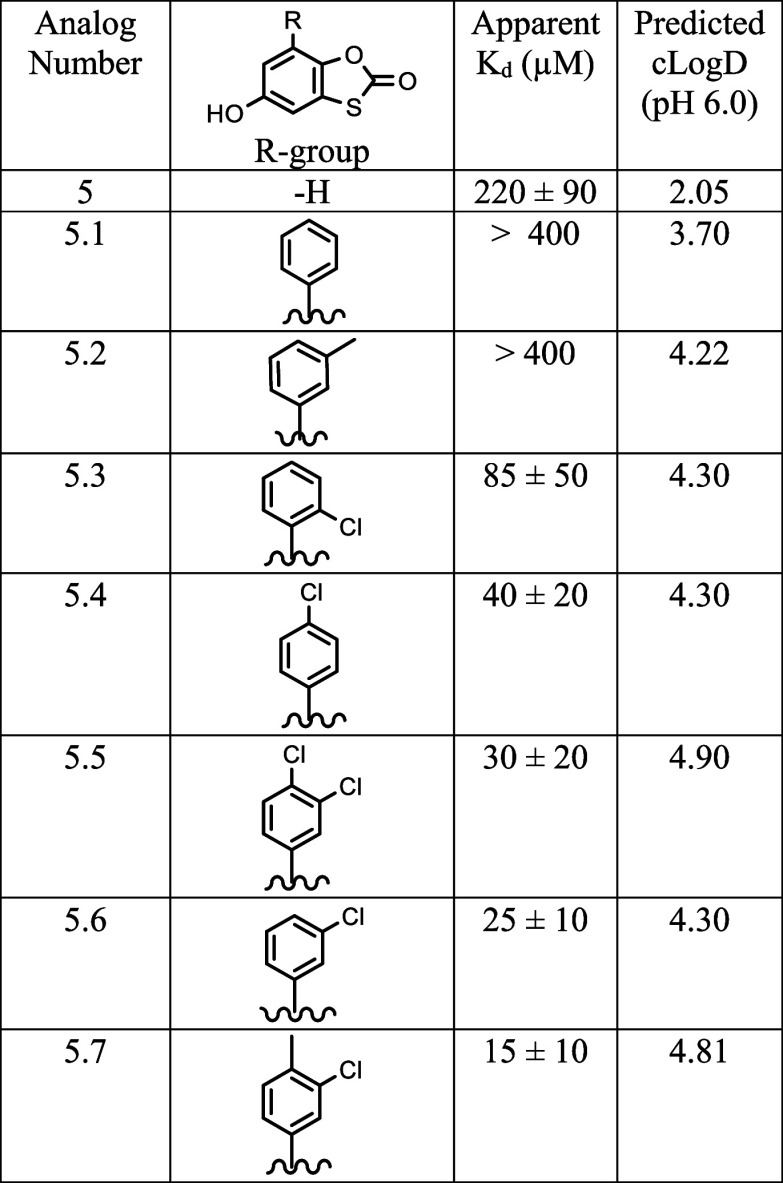
Apparent *K*_d_s and Predicted cLog *D*s of Titrated Analogues of Fragment 5^a^

a Standard error of global *K*_d_ fit of all selected residues is reported.

## Conclusions

Presented here is a new methodology that enables screening of small molecules and fragments for PMPs while the protein is engaged in the membrane. Use of mmRMs proves to eliminate some of the disadvantages of using other membrane models for fragment screening of membrane-engaged targets. This methodology allowed for the discovery of nine fragment ligands and highlights the ligandability of GPx4 in its membrane-bound state, contrasting the water-solubilized state which is considered unligandable by noncovalent inhibitors. These fragments may be used as building blocks for the development of noncovalent small-molecule inhibitors for GPx4, which have not yet been reported. The fragments span a broad range of partitioning coefficients, an advantage of using the mmRM model and its bulk-nonpolar environment. Partitioning properties were investigated using [^1^H–^1^H] NOESY experiments to reveal that regardless of where the fragments prefer residing, they can target and bind GPx4. Additionally, the potential to enhance and build off these fragments was shown through a SAR study. A secondary screen of fragment 5 analogues revealed a series of compounds with higher affinity, revealing a potential pathway toward developing inhibitors.

Importantly, these fragment hits would not have been discovered in the absence of a membrane model, demonstrating that GPx4 is more tractable in its active, membrane-bound form. Regardless of the platform, mmRM or micelle, direct targeting of the protein was not observed unless GPx4 was engaged with a membrane model. Our results suggest that the presence of a small-molecule binding site may depend on membrane engagement.^6^ The binding site that the fragments engage with may be due to a confirmational change upon membrane binding, or perhaps, the local environment of the protein surface that is solvated by lipids may be uniquely poised to bind these fragments. Further study is needed to reveal the details.

The versatility of the mmRM system in encapsulation of PMPs with varied topology and function suggests that this strategy may be applied to other PMP targets.^25,26^ There may also be utility for this screening technology for transmembrane proteins to target the nonsolvated parts of the proteins. RMs are known to house transmembrane proteins efficiently, allowing this as a potential avenue for screening these difficult targets.^35,63^ This method may be applied to MPs broadly and can be added to the repertoire of FBDD methodologies used to efficiently probe chemical space,^23,24^ take into account inhibitor–lipid interactions,^7^ assess ligandability,^14^ and initiate drug discovery endeavors with a better consideration of membrane partitioning properties.^6^ Often, drug design and development relies on the assumption that there are only nonspecific interactions within the membrane, making them unexploitable.^1^ There has been an increasing number of membrane protein structures with binding sites displaying displacement of membrane lipids upon ligand binding, indicating tractability in the membrane interface.^6,64,65^ All of these considerations point to the pressing need to expand the use of membrane-based screening platforms to ensure that the largest set of tools is available for these challenging targets.

## Methods and Materials

Methods for micelle, bicelle, and mmRM construction; DLS measurements; and GPx4 protein production were performed as previously reported.^25,52^ Details can be found in the Supporting Information.

### Protein ^15^N–^1^H HSQC

NMR samples were prepared with ^15^N-isotopically labeled protein with 10% v/v of *d*-pentane as the lock solvent (Sigma-Aldrich, St. Louis, MO). All NMR spectra were collected at 25 °C on a 700 MHz Bruker Avance III instrument. All NMR spectra were processed using NMRPipe^66^ and analyzed using NMRFAM-Sparky.^67^ Chemical shifts for GPx4 (BMRB 50955) were assigned from those previously published.^52^ Chemical shift perturbations from ^15^N-HSQC spectra were calculated using the following formula
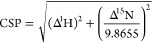
where Δ^1^H and Δ^15^N are the changes in ^1^H and ^15^N chemical shifts, respectively.

#### Fragment Delivery and Screening

A custom subset of 1911 members of the Life Chemicals high-solubility fragment library was used for this study. The subset members were selected to prioritize chemical diversity while still adhering to fragment rule-of-three metrics, to include only fragments with measured solubility confirmed at 1 mM in PBS, and to avoid PAINS compounds.^18,56^ To conduct the fragment screening, mixtures of 10 fragments were pipetted into individual vials for a total concentration of 400 μM for each fragment. Samples of mmRM-encapsulated GPx4 were transferred to the vials of predried fragments, an additional 50 μM of hexanol was added, and subsequent vortexing and water bath sonication ensured that the dried fragment was incorporated into the mmRM. The ∼190 mixtures of 10 fragments apiece were analyzed by ^15^N-HSQC experiments of GPx4. Spectra of each fragment mixture were compared to ^15^N-HSQCs of encapsulated GPx4 containing no fragment. Any mixture showing promising chemical shifts in the membrane-interacting surface was flagged as potentially containing a hit. From there, the best 15 mixtures were prioritized into three groups of five mixtures, which we termed high-priority, medium-priority, and low-priority. The five highest priority mixtures produced spectra with significant shifting with multiple resonances in the interface. Medium priority mixtures had less overall shifting but still contained multiple resonances of interest, while low priority mixtures had minimal shifting with 1–2 resonances of interest. It is important to note that this ranking system, while incorporating certain benchmarks, was qualitative and performed by the eye. Moving forward, for the sake of efficiency, only the 10 high and medium priority mixtures were progressed forward to the deconvolution stage.

Fragment members of the top 10 hit mixtures were tested individually to reveal the identity of the hit within each mixture. 400 μM fragment was encapsulated with GPx4 and compared against the ^15^N-HSQC spectrum of GPx4 in the mmRM without any fragment. CSP cutoffs were established to isolate the hits from the nonhits with a minimum of 7 out of the 15 cationic patch or catalytic site resonances producing CSPs greater than 0.01 ppm denoting a hit. Fourteen fragments were identified as GPx4 binders using the CSP cutoffs.

#### Fragment and Analogue Titrations and Aqueous Screen

The 14 fragments isolated from deconvolutions were purchased from Life Chemicals (fragments 1, 4, 6, and 11, and 12–14), Combi-Blocks (2, 3, 5, 9, and 10), or ChemBridge Hit2Lead (7 and 8). All fragment 1 and 5 analogues were also purchased from ChemBridge Hit2Lead. Fragments and analogues purchased from Life Chemicals and Hit2Lead are 90+% pure, while the fragments purchased from Combi-Blocks ranged from 95 to 97% pure. The day prior to the titration, a double-volume sample of encapsulated GPx4 was made before being split up into two separate vials. Half of the sample was added to the vial containing predried fragment corresponding to 400 μM or 1 mM. The following day, the sample lacking fragment was used as the zero point of the titration and was compared to the high point, 400 μM or 1 mM of fragment, prior to the start of the titration to evaluate if significant CSPs were observed in the membrane interface region. If they were, then the two samples were mixed to produce varying concentrations and to measure a titration curve. The top-shifting resonances were determined by calculating the average CSP at 400 μM or 1 mM and 1 and 2 standard deviations (σ) above that average. The CSP for the resonance at each fragment concentration was extracted and fit to a curve using an affinity equation that accounts for ligand depletion
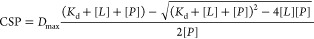


Any resonance producing a curve with an *R*^2^ below 0.85 was removed, and the remaining resonances were then used to fit a global apparent *K*_d_ curve for the fragment hit. Standard error for *K*_d_ from the global fit of all of the selected residues is reported.

Fragments 1, 5, and 6 were selected to perform proof-of-concept experiments in bulk aqueous conditions. 400 μM and 15 mM aliquots of fragment were vacuum-dried overnight to remove excess DMSO. An apo GPx4 reference was collected with 1% DMSO or 5% DMSO to ensure that CSP calculations took into account DMSO needed for fragment solubilization. 100 μM GPx4 was added to the dried fragments, and DMSO was added to a total of 1% for the 400 μM and 5% for the 15 mM fragment experiments. These percentages were the lowest amounts needed to solubilize the fragments in solution. Prior to experimentation, the pH of the sample was checked to ensure that no change was observed upon the addition of the fragments. However, the 100 mM Bis-Tris at pH 6.0 ensured that the pH was not altered. ^15^N-HSQC experiments were collected, and CSPs were analyzed and compared to the top-shifting resonances determined from the mmRM experiments. DLS measurements were collected to assess fragment aggregation in the aqueous conditions using a Malvern Zetasizer Nano-S instrument. At 400 μM, the fragments were combined in buffer with 1% DMSO, and at 15 mM fragment, 5% DMSO was used to enhance solubility.

#### Fragment Partitioning by ^1^H–^1^H NOESY

Partitioning coefficients for fragment hits and analogues were calculated using a Marvin Log *D* calculator (Chemaxon) and were used to prioritize fragments for 2D NOESYs. mmRMs were premade as previously described and loaded with 5 mM dried fragment 1, 5, or 6 before shaking overnight. A high concentration of fragments is needed for these experiments to observe the intermolecular NOEs between the fragments and the membrane model components. For the DHPC/DMPC samples, the *q* = 1.0 bicelles were constructed routinely,^55^ as described in the Supporting Information, with an additional step to ensure the complete removal of water before experimentation. An aliquot of buffer (100 mM Bis-Tris pH 6.0, 100 mM NaCl, and 10 mM DTT) was frozen and freeze-dried overnight to remove residual water. The dried buffer was then resuspended in 100% D_2_O, mixed, and frozen before another round of freeze-drying. The components were then resuspended in 100% D_2_O again before being incorporated into the DHPC fraction of the bicelle. The DHPC solution was then mixed with dried DMPC, and bicelle formation proceeded. The NOESY experimental design was based on a previous work.^60^ All NMR spectra were collected at 25 °C on a 700 MHz Bruker Avance III instrument. The 2D data was collected with a 90° pulse width between 10.2 and 11.0 μs, which was optimized prior to each experiment. 128 complex increments were collected in the State-TPPI mode with 8 scans per increment, 2 s recycle time, and 250 ms mixing time. All spectral data was processed with NMRPipe^66^ and analyzed with NMRFAM-Sparky.^67^

## Supporting Information Available

Methods for micelle, bicelle, and mmRM preparations; methods for dynamic light scattering; methods for protein expression and purification; compatibility of membrane models for fragment screening by DLS; fragment hit characterizations by NMR titration; CSP analysis of GPx4 embedded in mmRMs and bound to fragments; full [^1^H–^1^H] NOESY spectra of fragments in mmRMs; full [^1^H–^1^H] NOESY spectra of fragments in bicelles; apparent *K*_d_s and *c* Log *P*s of titrated analogues of fragment 1; CSPs of mmRM-embedded GPx4 upon addition of analogue 5.5; and references (PDF)
